# Integratome analysis of adipose tissues reveals abnormal epigenetic regulation of adipogenesis, inflammation, and insulin signaling in obese individuals with type 2 diabetes

**DOI:** 10.1002/ctm2.596

**Published:** 2021-12-19

**Authors:** Nana Jin, Heung‐Man Lee, Yong Hou, Allen C.S. Yu, Jing‐Woei Li, Alice P.S. Kong, Candice C.H. Lam, Simon K.H. Wong, Enders K.W. Ng, Ronald C.W. Ma, Juliana C.N. Chan, Ting‐Fung Chan

**Affiliations:** ^1^ School of Life Sciences The Chinese University of Hong Kong Hong Kong SAR China; ^2^ Department of Medicine and Therapeutics, The Chinese University of Hong Kong Prince of Wales Hospital Hong Kong SAR China; ^3^ Hong Kong Institute of Diabetes and Obesity, The Chinese University of Hong Kong Prince of Wales Hospital Shatin Hong Kong SAR China; ^4^ Li Ka Shing Institute of Health Sciences, The Chinese University of Hong Kong Prince of Wales Hospital Shatin Hong Kong SAR China; ^5^ Department of Surgery, The Chinese University of Hong Kong Prince of Wales Hospital Hong Kong SAR China

Dear Editor,

Obesity and type 2 diabetes (T2D) often but, not invariably, coexist. We examined the transcriptomes and methylomes of subcutaneous adipose (SAT) and visceral adipose tissues (VAT) samples from 25 obese individuals (36% men; mean±standard deviation (SD) body mass index: 39.1 ± 4.6 kg/m^2^; age: 38.6 ± 11.8 years; 12 had T2D; Table S1) with or without T2D during metabolic surgery. By integrating these datasets with public tissue‐specific regulatory networks, we revealed perturbations in adipogenesis, inflammatory, and insulin signaling pathways in obese individuals with T2D with validation using multiple external databases.

The whole transcriptome profiles identified only a few differentially expressed genes (DEGs) in T2D, particularly in T2D‐SAT (Figure 1A; Table S4). This finding accorded with previous reports showing only modest differences in gene expression between T2D and control subjects^1^. Other studies also identified DEGs implicated in glucose and insulin metabolism mainly in VAT compared with SAT^2^. These low levels of expression emphasize the need to use an integrated approach to identify these complex gene networks. Despite these differences in DEG in SAT and VAT, enrichment analysis indicated dysregulation of cell metabolism and inflammation in both SAT and VAT in obese individuals with T2D (Figure 1B,C).

**FIGURE 1:**
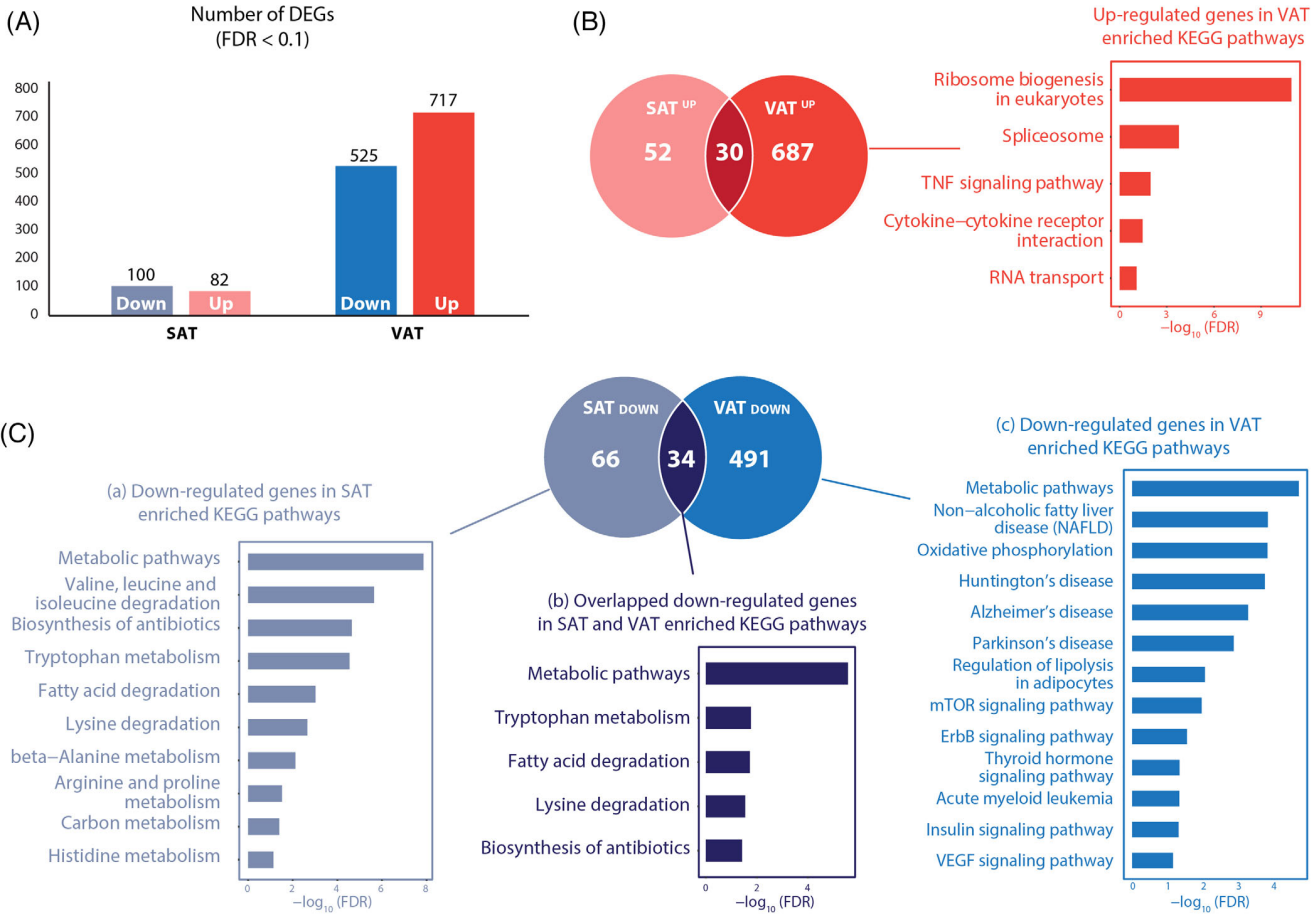
Whole transcriptome profiling reveals dysregulated cell metabolism and increased inflammation in obese individuals with T2D. (A) Number of DEGs (FDR<0.1) in the T2D versus control groups in SAT and VAT. (B) Number of upregulated genes in SAT and VAT. Enriched KEGG pathways (FDR<0.1) of upregulated genes in T2D in VAT. (C) Number of downregulated genes in SAT and VAT. Enriched KEGG pathways (FDR<0.1) of downregulated genes in T2D in SAT and VAT.

Global methylation levels, as determined by their relative distances to CpG islands, transcription start sites (TSSs), histone marks, enhancer regions, and other annotations, were similar between VAT and SAT in both T2D and control individuals (Figure S2A‐E). However, transcription factor binding sites (TFBSs) in T2D‐VAT showed a differential distribution of methylation compared to T2D‐SAT (Figure 2A,B). Additionally, 19 and 31 differentially methylated regions (DMRs) were detected in T2D‐SAT and T2D‐VAT, respectively (Figure 2C; Table S5). We discovered a novel hypomethylated region in the promoter of *LCLAT1* in both T2D‐VAT and T2D‐SAT samples (Figure 2D). According to ENCODE data and ChromHMM analysis, this hypomethylated region could facilitate transcription factor (TF) binding and activate gene expression. We also found hypermethylation spanning the 5’UTR of *HOXA3* specific to T2D‐SAT, accompanied by a depletion of its coding mRNA levels in individuals with T2D (Figure 2E).

**FIGURE 2:**
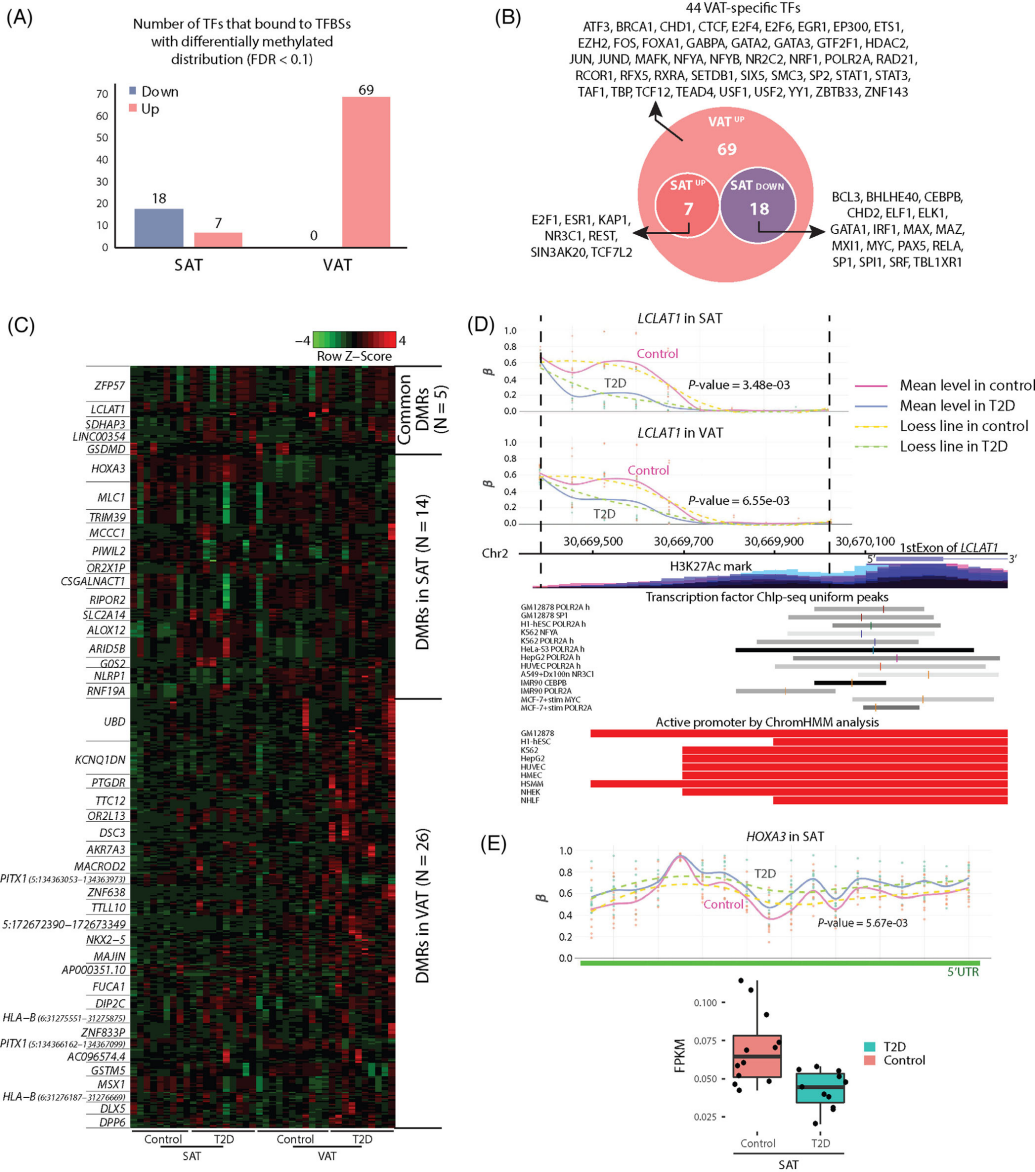
Whole methylome profiling of TFBSs and DMRs in VAT and SAT in obese individuals with T2D (A) Number of TFs that bind to TFBSs with differential methylation (FDR<0.1). (B) Venn diagram showing the number of TFs that bind to TFBSs with similar, divergent, or tissue‐specific differential methylation in SAT and VAT. (C) Heat‐map of the methylation levels of five common DMRs in SAT and VAT, 14 SAT‐specific DMRs, and 24 VAT‐specific DMRs. Rows and columns represent methylation probes and samples, respectively. (D) Methylation levels and histone modifications of *LCLAT1*. Novel hypomethylated regions in the promoter (TSS1500) region of *LCLAT1* in SAT and VAT. Histone modifications in the hypomethylated regions of *LCLAT1* were determined using ENCODE data. (E) Methylation levels and expression of *HOXA3* in SAT. Novel tissue‐specific hypermethylated regions in the 5’UTR of *HOXA3* in SAT. Boxplot showing FPKM of *HOXA3* in T2D‐SAT and control‐SAT. Colors represent disease groups (T2D; control).

Simply integrating DEGs with DMRs cannot fully elucidate the complex biological networks implicated in T2D and obesity. Thus, we used tissue‐specific regulatory networks to discover epigenetically dysregulated gene modules in adipose tissues and their associations with T2D (Supplemental materials). We detected three modules in T2D‐SAT and five modules in T2D‐VAT by integrating transcriptomes, methylomes, and tissue‐specific regulatory networks (Figure 3A, 3C, 3E; Figure S3A‐F). There were 19 genes common to the T2D‐SAT and T2D‐VAT modules, with an enriched functional annotation of transcriptional regulation (Figure 3B). These findings suggested dysregulated biological pathways shared by SAT and VAT in obese individuals with T2D.

**FIGURE 3:**
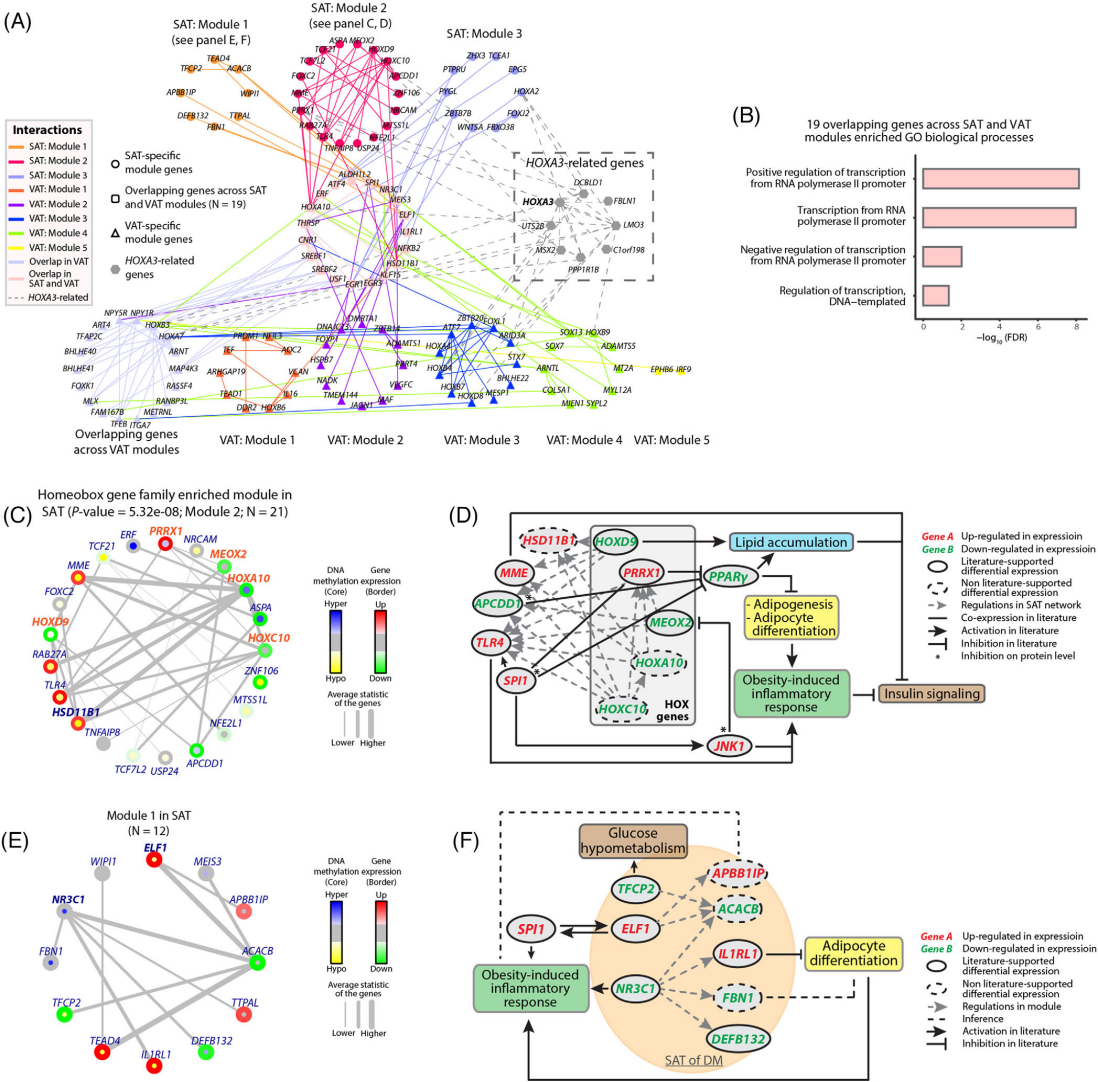
Tissue‐specific functional epigenetic network‐based analysis of SAT and VAT. (A) Overview of the three and five functional epigenetic modules identified in SAT and VAT, respectively. The significance of the colors of the nodes and edges are described in the legend. The 19 genes in the grey frame are the genes shared between SAT and VAT. Dashed lines represented regulatory interactions between *HOXA3*‐related genes and module genes in the SAT‐specific network. (B) Enriched GO biological processes (FDR<0.1) of the 19 shared genes, including transcriptional regulation‐related biological processes. (C) Homeobox gene family‐enriched module in SAT (Module 2). Genes in orange represent the homeobox gene family genes. Edge widths represent the average statistic of the genes comprising the edge. The core of the node represents the differential DNA methylation statistics. The border of the node represents the differential gene expression statistics. Regulatory direction is not shown. (D) Literature‐based interpretation of how *HOX* genes and their targets identified in SAT module 2 can lead to dysfunction of adipogenesis and adipocyte differentiation, thereby activating obesity‐induced inflammatory response and inhibiting insulin signaling. Edge and node colors are explained in the legend. (E) Tissue‐specific functional epigenetic module 1 in SAT. (F) Literature‐based interpretation (solid line) of genes in the tissue‐specific SAT module that lead to obesity‐induced inflammatory responses and the inference (dashed line) of the module function.

Among the 82 T2D‐VAT module genes, some were implicated in circadian rhythm, disruption of which could contribute to the development of T2D^3^, while many others were known genes associated with T2D. For example, slight downregulation of *TFEB* might result in reduced adipogenesis known to be associated with an increased risk of T2D^4^.

Interestingly, we identified a *HOX* gene‐enriched module in T2D‐SAT (Figure 3C‐D, Supplemental materials). The distribution and pattern of *HOX* genes differed between the upper and lower body which might explain the different prognostic significance of VAT (predominate in the upper body) and SAT (predominate in the lower body)^5^. Given the association of VAT with cardiovascular disease and T2D risk, and the protective effects of SAT^6^, the identification of *HOX* genes as a major linking biomarker provides new insights regarding the causal role of adipogenesis in T2D.

In this *HOX* gene‐enriched T2D‐SAT module, we identified multiple genes with differential expression and methylation, with several examples highlighted (Figure 3D). Consistent with the previous studies,^7^ we found downregulated trends of *HOXD9* and *MEOX2*, and upregulation of *PRRX1*. Along with *APCDD1* and *SPI1, PRRX1* could inhibit PPARγ‐mediated adipocyte differentiation and adipogenesis. On the other hand, cooperative expression of *HOXD9*, *MME*, *SPI*, and *TLR4* might impair insulin signaling and secretion accompanied by obesity‐induced inflammatory responses^8^, ^9^. Using EpiMap^10^, the rs34872471 genetic signal overlapped with the adipose tissue‐specific enhancer region nearest to the *TCF7L2* promoter in T2D patients (Figure 3C). Other genes in the module were potentially novel T2D markers, such as *ASPA*, an interactor of *TCF7L2*, which was hypermethylated with downregulation in T2D‐SAT. The novel T2D‐SAT‐specific 5’UTR of *HOXA3* hypermethylated region (Figure 2E) was related to all *HOXA3*‐regulated genes (Figure 3A), supporting their roles in the epigenetic regulation in T2D. Taken together, this *HOX* gene‐enriched module may participate in inhibiting PPARγ‐mediated adipocyte differentiation and adipogenesis, and impairing insulin signaling and secretion accompanied by obesity‐induced inflammatory responses.

In another T2D‐SAT‐specific module (Figure 3E‐F), we identified novel T2D biomarkers. The expression levels of *ACACB*, *ELF1*, *IL1RL1*, and *SPI1* were confirmed by qPCR validation in additional T2D‐SAT samples (Figure S4). *ELF1*, *IL1RL1, NR3C1*, and *TFCP2* were known to reduce adipocyte differentiation which can lead to abnormal glucose metabolism and inflammation, while *APBB1IP* and *FBN1* were predicted to be involved in these biological pathways. Taken together, this module might provide a novel epigenetic pathway regulating insulin signaling through adipocyte differentiation and inflammatory responses in obese patients with T2D.

We identified 161 potential biomarkers in these networks and DMRs which were independently validated in at least one external database relevant to comorbidity, druggability, expression quantitative trait loci (eQTL), genome‐wide association studies (GWAS), TFBSs, TFs, or the T2D integratome (T2Di) (Figure 4A‐B; Table S6‐S7). Of these 161 modular biomarkers enriched in multiple databases, 73.9% were validated in at least one external dataset and 48.4% were TFs. Amongst the biomarkers shared by both tissues, 7 were potential drug targets.

**FIGURE 4:**
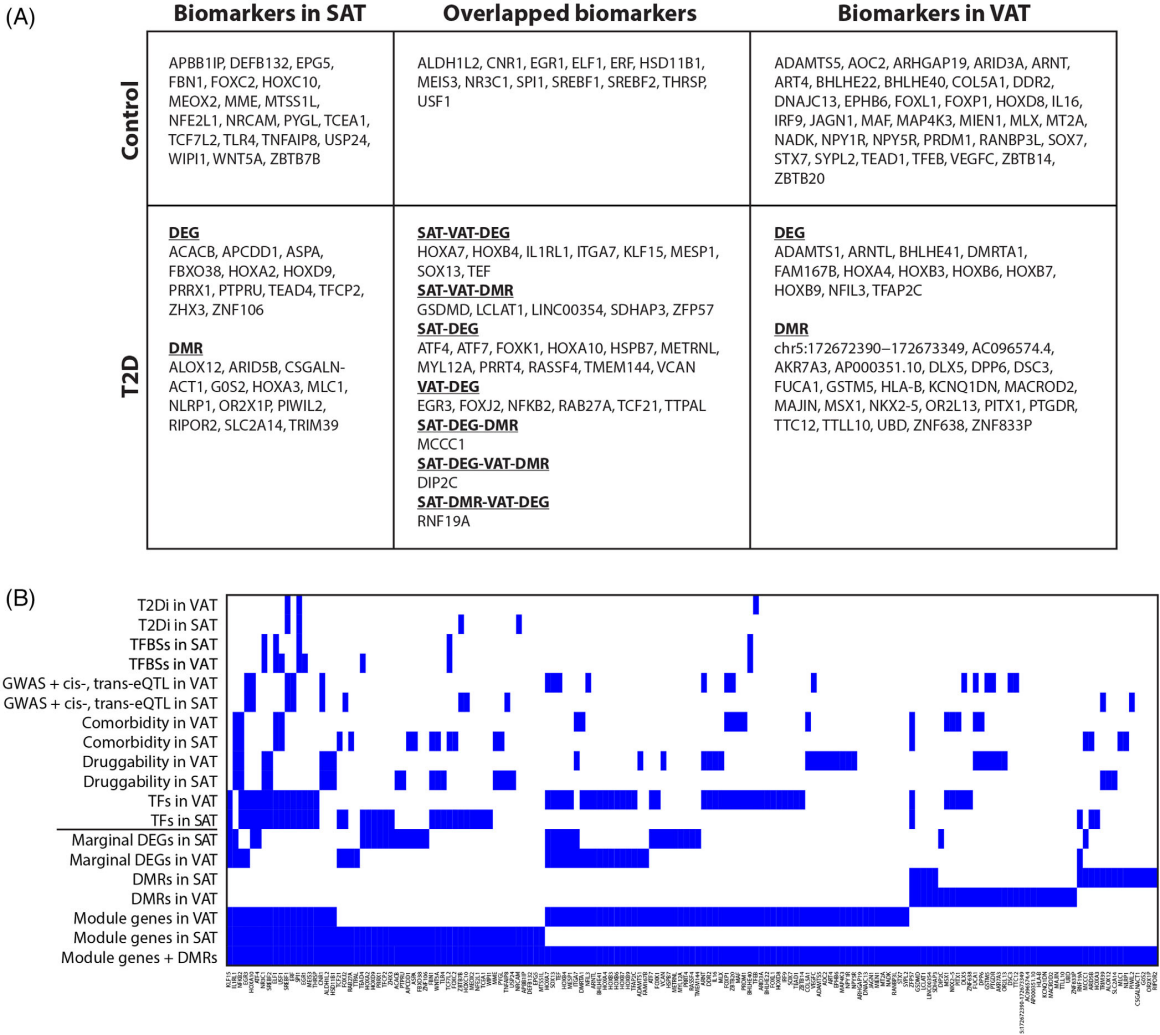
(A) Summary of the 161 module‐ and DMR‐identified biomarkers in T2D and control SAT and VAT. DEGs represent marginal DEGs. T2D biomarkers are those differentially expressed or methylated genes in T2D. Control biomarkers are those that interacted with the T2D biomarkers, but no differential expression or methylation in T2D. (B) Datasets used for external validation of module genes and DMRs in this study (i.e., comorbidity, druggability, eQTL, trans‐ethnic GWAS, TFBSs, TFs, and T2Di datasets).

By integrating differential gene expression and methylation levels in SAT and VAT collected during metabolic surgery from obese T2D and non‐T2D individuals with tissue‐specific regulatory networks, we found multiple epigenetic regulatory networks in both SAT and VAT associated with obesity in T2D. These findings confirmed current knowledge regarding the pathophysiological roles of different adipose tissues in insulin resistance, inflammation, and development of T2D whilst revealing novel relationships not detectable by single‐layered analysis.

## CONFLICT OF INTEREST

ACSY, JWL are co‐founders of Codex Genetics Limited. NJ is working at Codex Genetics Limited. JCNC and RCWM are co‐founders of GemVCare.

## ETHICAL APPROVAL AND CONSENT TO PARTICIPATE

The research protocol and informed consent forms were approved by the Joint Chinese University of Hong Kong‐New Territories East Cluster Clinical Research Ethics Committee (Ref no. CRE‐2011.131) in accordance with the Declaration of Helsinki. All individuals gave written informed consent.

## AUTHOR CONTRIBUTIONS

TFC, JCNC, and RCWM conceived and directed the study. APSK and CCHL managed the clinical research ethics and patient consent. SKHW and EKWN performed the adipose tissue biopsies. HML and YH performed the experiments. NJ, ACSY, and JWL performed the bioinformatic analyses. NJ, TFC, and JCNC interpreted the data. NJ drafted the manuscript. TFC and JCNC revised the manuscript. All authors read and approved the final version.

## Supporting information

Supplementary Figure S1. Quality control for RNA and DNA samples. (A) 5’‐3’ bias detection for RNA‐Seq data in T2D and control as well as SAT and VAT samples separately. (B‐H) Signal distribution of quality control probes across all samples, including the efficiency of staining, hybridization, extension, and bisulfite conversion steps, as well as monitoring allele‐specific extension.

Supplementary Figure S2. Associations with epigenetic markers. (A) Global methylation levels with respect to the relative distance to CpG islands. (B) Global methylation levels with respect to the relative distance to the nearest TSS. (C) Global methylation levels according to gene expression level and the distance to the nearest TSS. (D) Global methylation levels of regions overlapping with FANTOM5 enhancers. (E) Global methylation levels of regions overlapping with ENCODE‐clustered histone modification peaks.

Supplementary Figure S3. Tissue‐specific functional epigenetic modules in SAT and VAT. (A) Tissue‐specific functional epigenetic module 3 in SAT. (B–F) Tissue‐specific functional epigenetic modules in VAT. Edge widths represent the average statistics of the genes making up the edge. The core of the node represents the differential DNA methylation statistics. The border of the node represents the differential gene expression statistics.

Supplementary Figure S4. qPCR validation of novel SAT‐specific markers associated with inflammation in obese individuals with T2D. Results were normalized to the gene expression levels of GAPDH. The gene expression differences between the T2D and the non‐T2D groups were compared using the Student's t‐test and the *p‐*values are indicated.

Supporting Information

Supporting Information

Supporting Information

Supporting Information

Supporting Information

Supporting Information

Supporting Information

Supporting Information

## Data Availability

RNA sequencing data are deposited at the NCBI Sequence Read Archive (SRA) database with the accession number PRJNA679994. DNA methylation data are deposited at the Gene Expression Omnibus (GEO) database with the accession number GSE162166.
