# Supplementary figures and images for: Integratome analysis of adipose tissues reveals abnormal epigenetic regulation of adipogenesis, inflammation, and insulin signaling in obese individuals with type 2 diabetes
Source: Clin Transl Med. 2021 Dec 19;11(12):e596. doi: 10.1002/ctm2.596 (PMC8684766; doi:10.1002/ctm2.596)

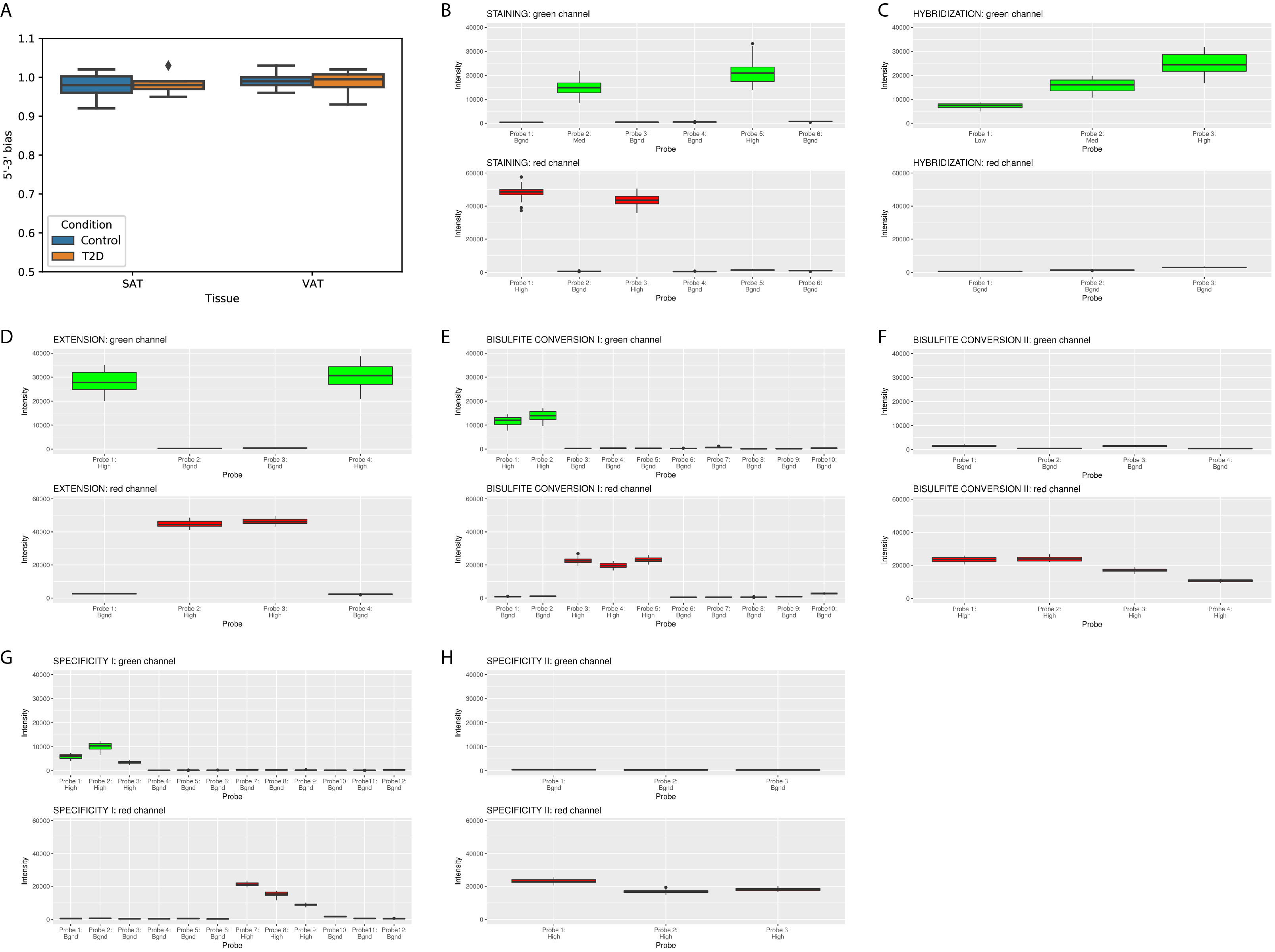

Supplement: Supplementary file 1 — Supplementary Figure S1. Quality control for RNA and DNA samples. (A) 5’‐3’ bias detection for RNA‐Seq data in T2D and control as well as SAT and VAT samples separately. (B‐H) Signal distribution of quality control probes across all samples, including the efficiency of staining, hybridization, extension, and bisulfite conversion steps, as well as monitoring allele‐specific extension. [file CTM2-11-e596-s010.jpg]

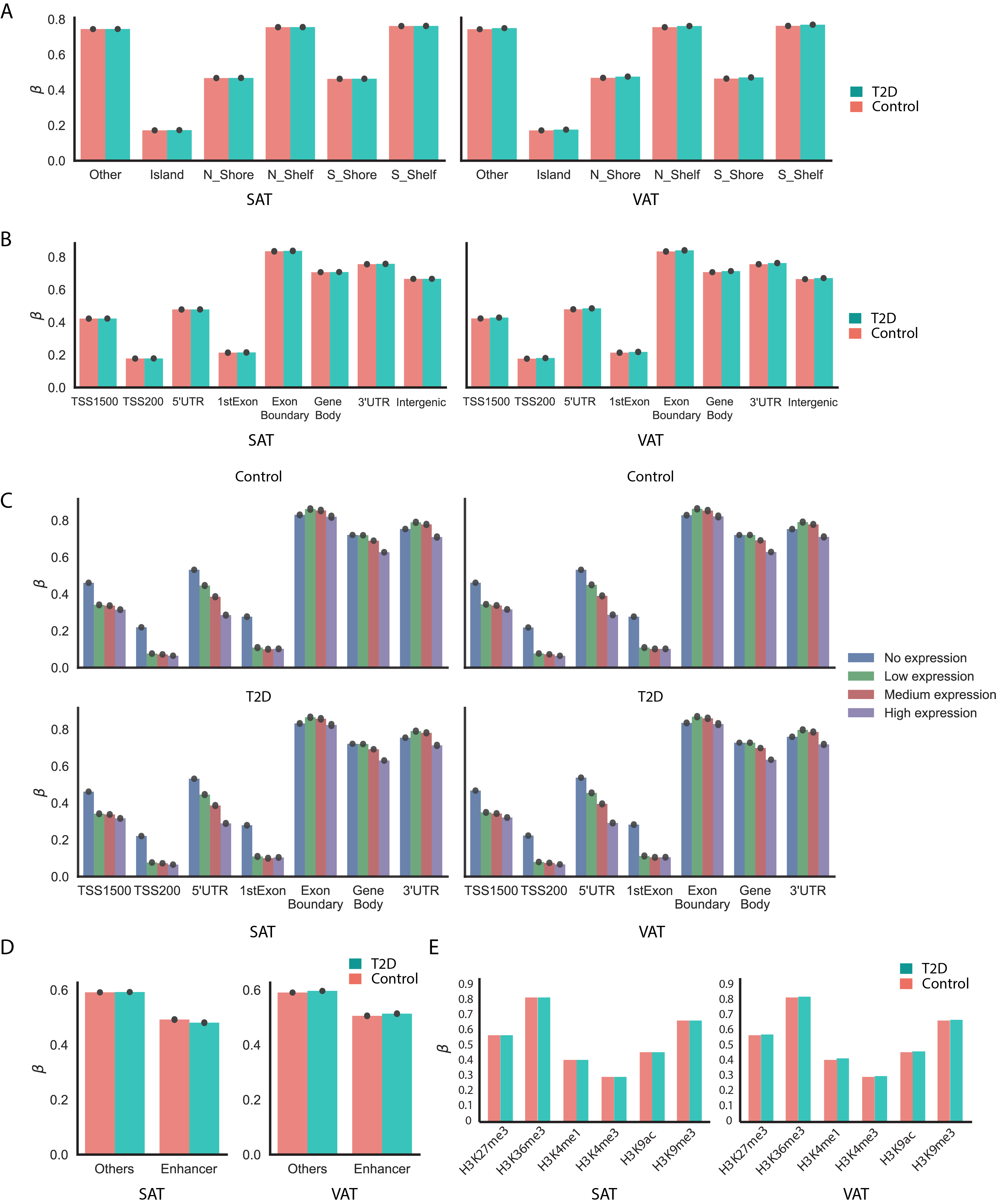

Supplement: Supplementary file 2 — Supplementary Figure S2. Associations with epigenetic markers. (A) Global methylation levels with respect to the relative distance to CpG islands. (B) Global methylation levels with respect to the relative distance to the nearest TSS. (C) Global methylation levels according to gene expression level and the distance to the nearest TSS. (D) Global methylation levels of regions overlapping with FANTOM5 enhancers. (E) Global methylation levels of regions overlapping with ENCODE‐clustered histone modification peaks. [file CTM2-11-e596-s001.jpg]

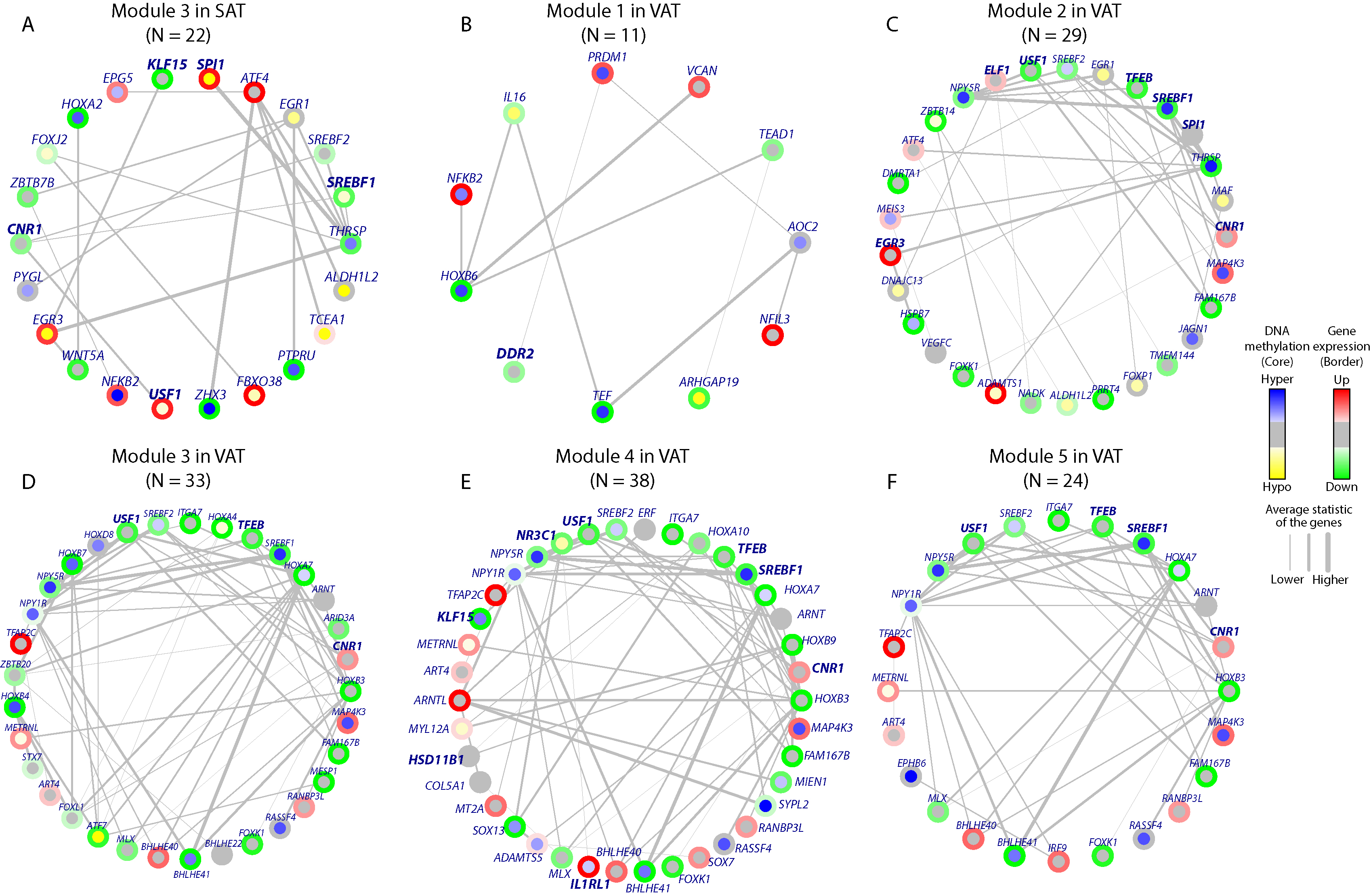

Supplement: Supplementary file 3 — Supplementary Figure S3. Tissue‐specific functional epigenetic modules in SAT and VAT. (A) Tissue‐specific functional epigenetic module 3 in SAT. (B–F) Tissue‐specific functional epigenetic modules in VAT. Edge widths represent the average statistics of the genes making up the edge. The core of the node represents the differential DNA methylation statistics. The border of the node represents the differential gene expression statistics. [file CTM2-11-e596-s009.jpg]

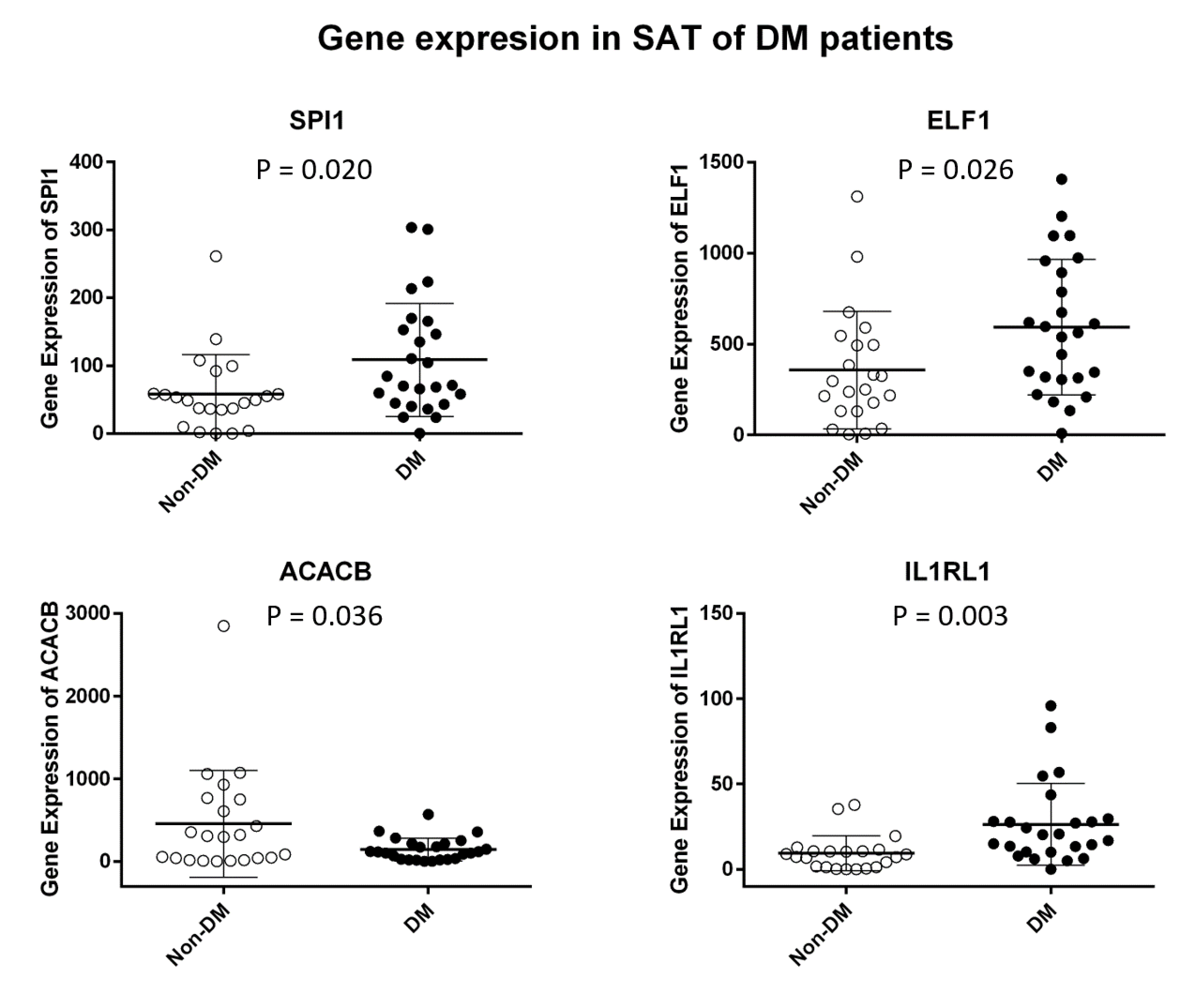

Supplement: Supplementary file 4 — Supplementary Figure S4. qPCR validation of novel SAT‐specific markers associated with inflammation in obese individuals with T2D. Results were normalized to the gene expression levels of GAPDH. The gene expression differences between the T2D and the non‐T2D groups were compared using the Student's t‐test and the p‐values are indicated. [file CTM2-11-e596-s004.jpg]
