# Supplementary material for: Integratome analysis of adipose tissues reveals abnormal epigenetic regulation of adipogenesis, inflammation, and insulin signaling in obese individuals with type 2 diabetes
Source: Clin Transl Med. 2021 Dec 19;11(12):e596. doi: 10.1002/ctm2.596 (PMC8684766; doi:10.1002/ctm2.596)
Supplement: Supplementary file 12 — Supporting Information [file CTM2-11-e596-s011.docx]

**Methods and materials**

**Subject recruitment**

The Prince of Wales Hospital (PWH) is the teaching hospital of the Chinese University of Hong Kong (CUHK) and a regional hospital that serves a population of over 1.2 million. The Multidisciplinary Clinic of Metabolic and Bariatric Surgery at PWH is jointly run by the PWH and the Department of Surgery, CUHK, and targets individuals with morbid obesity (BMI > 30 kg/m^2^) for weight reduction procedures. All individuals who agreed to undergo surgery were invited to donate tissues (subcutaneous adipose tissue (SAT) and visceral adipose tissue (VAT)) for research and publication purposes with written informed consent prior to their operation. The adipose tissue was collected after successful completion of all major procedures and prior to closing the incision. The omental fat was collected as the VAT and the subcutaneous fat close to the incision site was collected as SAT. The tissue collected was immediately snap-frozen in liquid nitrogen and stored at -70°C until RNA and DNA extraction were performed.

The research protocol including informed consent forms were approved by the Joint Chinese University of Hong Kong–New Territories East Cluster Clinical Research Ethics Committee (Ref no. CRE-2011.131) in accordance with the Declaration of Helsinki. All individuals gave written informed consent.

All individuals included in our analysis were not related to each other. All individuals underwent a detailed clinical assessment including a 75 g oral glucose tolerance test in those without a known history of type 2 diabetes (T2D) to establish their glycemic status. Diagnosis of T2D was based on the American Diabetes Association (ADA) criteria. Between January 2012 and August 2014, 12 adults with T2D and 13 without T2D (control) participated in the study (Supplementary Table S1).

**DNA and RNA extraction**

RNA samples were prepared for sequencing using the RNeasy Mini Kit (Qiagen, Germany) according to standard protocols, with in-column DNase treatment to avoid genomic DNA contamination. All sequenced RNA had a 260/280 ratio of ≥1.9 and a bioanalyzer was used to confirm that ribosomal RNA peaks were intact and that the samples had an RNA integrity number (RIN) value > 7.2. RNA was extracted from SAT samples for validation of gene expression using TRIzol reagent (ThermoFisher, USA) according to standard procedures. All RNA samples used for validation had a 260/280 ratio ≥1.8. First strand cDNA synthesis was carried out using a High-Capacity cDNA Reverse Transcription Kit (ThermoFisher, USA) with 200 ng of total RNA. All procedures were carried out according to the manufacturer’s standard protocols. Total DNA samples were prepared with Proteinase K digestion and phenol extraction. All DNA samples had a 260/280 ratio ≥ 1.8.

**RNA sequencing**

We applied paired-end sequencing libraries to 44 RNA sequencing samples (RNA-Seq) from 25 individuals. Of these 44 samples, the SAT samples were from 11 T2D and 12 control individuals, while the VAT samples were from 10 T2D and 11 control individuals. Sequencing reads were dynamically trimmed using Trimmomatic v.0.32 [1]. Quality trimmed reads were mapped onto the human genome (hg19) by STAR v.2.3.0e [2]. QualiMap (v2.2.2) was used to check with the quality for RNA samples [3]. We used Cufflinks v.2.2.1 [4] to calculate Fragments Per Kilobase of transcript per Million mapped reads (FPKM) of genes overlapping with genes annotated in Ensembl Release 75. To minimize bias, data from different tissues were analyzed separately. We used the Kruskal-Wallis rank-sum test to examine the confounding effect of gender, and linear regressions to examine the effects of age, BMI, waist and hip measurements on expression levels (Supplementary Table S2). DEGs were detected by the R/Bioconductor package *limma* (v3.30.11) [5]. To optimize the detection of DEGs with low expression levels, we only included genes with FPKM >0.1 prior to DEG analysis. Kyoto Encyclopedia of Genes and Genomes (KEGG) pathway [6] and Gene Ontology term [7] enrichment was performed using DAVID (v6.8) [8].

**MethylationEPIC BeadChip**

Forty methylation samples from 24 individuals were sequenced. Of these samples, SAT samples were from 10 T2D and 9 control individuals and VAT samples were from 10 T2D and 11 control individuals. Bisulfite conversion of the DNA was conducted using the EZ DNA Methylation Kit (Zymo Research). DNA methylation was quantified using an Infinium MethylationEPIC BeadChip kit (Illumina, CA) run on an Illumina iScan System (Illumina) as per the manufacturer’s instructions.

**Methylome pre-processing**

The Illumina EPIC array was used to assess methylated sites across the whole genome for quantifying the methylomes of the 19 SAT and 21 VAT samples. Several filters were used to remove probes that could potentially bias the results using the R/Bioconductor package *RnBeads* (v1.7.1). First, to reduce bias in probe-binding efficiency due to differences in individual genetic makeup, probes overlapping with SNPs in the last three bases of the target sequence were removed [9]. Second, the Greedycut algorithm [10] was used for the iterative removal of probes that produced unreliable measurements. Third, probes that did not target CpG sites or were located on sex chromosomes were removed. The remaining probes were subjected to background correction using the ENmix method [11] and normalized using the beta-mixture quantile normalization method [12].

To assess the number and nature of the significant components of variation in SAT and VAT methylation profiles, the singular value decomposition (SVD) method was used (R/Bioconductor package *base* v3.5.1) [13]. The Kruskal-Wallis rank-sum test and linear regression analysis were applied to categorical and numeric data, respectively. The covariates included age, BeadChip Sentrix ID, BeadChip Sentrix Position, sex, and sample barcode (Supplementary Table S3). The BumpHunter method (v1.35.0) [14] was used to detect differentially methylated regions (DMRs). ChromHMM [15] analysis was used to characterize chromatin states. ChromHMM track was retrieved from UCSC genome browser on 4 Sep 2017.

**Tissue-specific regulatory networks**

We used tissue-specific regulatory networks published by Marbach *et al.* [16]. The directed and weighted networks were inferred by integrating transcription factor (TF) sequence motifs published by Kheradpour *et al.* [17] with tissue-specific promoter and enhancer activity data from the FANTOM5 project [18]. The tissue-specific regulatory network construction workflow involved: (1) genome-wide mapping of promoters and enhancers, (2) linking TFs to promoters and enhancers using TF-binding motifs and tissue-specific expression of target elements, and (3) linking enhancers and promoters to target genes based on genomic distance and joint expression in the given tissue. The edges of the networks were further validated using chromatin immunoprecipitation sequencing (ChIP-seq) from ENCODE [19], expression quantitative trait loci (eQTL) from GTEx [20] and RNA-Seq data from the Roadmap Epigenomics Project [21]. Specifically, we curated two tissue-specific regulatory networks, omental adipocytes for VAT and subcutaneous adipocytes for SAT, in which the detailed circuitry of enhancers and promoters were encapsulated [16].

There were 613,156 and 751,141 non-redundant TF-gene regulatory relationships, involving 12,350 and 12,639 TFs, detected in the SAT and VAT regulatory networks, respectively. The weights of these regulatory relationships were inferred using TF-binding motifs and tissue-specific expression of the target elements [16]. The top 10,000 regulatory relationships were used to construct tissue-specific regulatory networks for our study, incorporating 2,736 and 2,493 nodes in the SAT and VAT networks, respectively.

**Functional epigenetic modules (FEM) algorithm**

Epigenetically dysregulated gene modules are subnetworks that drive the expression of T2D-related genes through an underlying epigenetic mechanism. Such gene modules were detected using the functional epigenetic modules (FEM) algorithm (v3.10.0) [22]. This algorithm performs a supervised analysis to identify subnetworks by integrating DNA methylation levels and mRNA expression using tissue-specific regulatory networks as a scaffold. The methylation levels of probes targeting the same gene were averaged. Methylation data were derived from regions 200 bp upstream of the transcription start site (TSS200) within the first exon, or 1500 bp upstream of the transcription start site (TSS1500). As methylation levels are usually negatively correlated with gene expression, a module composed of genes with strong negative correlations between gene expression and DNA methylation were assigned a high module score [22]. To ensure that stable results were obtained, the number of permutations was set to 5,000. All the R/Bioconductor packages were installed on R with version higher than 3.

**External validation datasets**

Several external datasets were used to confirm the reproducibility of the biomarkers identified. These datasets included comorbidity, druggability, eQTL, trans-ethnic genome-wide association studies (GWAS), TFBSs, TFs, and the T2D interactome (T2Di) [23]. Disease-associated genes were obtained from the disease network constructed by Menche *et al.* [24]. Disease enrichment analysis of the biomarkers was performed using a hypergeometric test (*p* <0.01). Drug targets were identified using the Drug Gene Interaction Database (DGIdb, v3.0.2) [25] and Qiagen’s Ingenuity Pathway Analysis (IPA; Qiagen, USA; www.qiagen.com/ingenuity). eQTL data were retrieved from MuTHER [26] and GTEx [20]. GWAS data were obtained from the DIAbetes Genetics Replication and Meta-analysis (DIAGRAM) database [27]. The Sherlock statistical framework was used to identify potential disease-associated genes by matching eQTL signals with GWAS associations [28]. Transcription factors were retrieved from the Gene Ontology Consortium [29]. A T2Di developed by our group using transcriptome data and public databases [23] was also used.

**qPCR validation**

Forty-seven SAT RNA samples were used to validate the expression of various modular genes. qPCR was carried out using the SYBR Green method with the QuantStudio 12K Flex Real-Time PCR System and GoTaq Master Mix (Promega, USA). Results were normalized to the gene expression levels of GAPDH. Gene expression was compared between the T2D and the non-T2D groups with the Student’s t-test. Data are presented as mean ± standard deviation (SD), unless otherwise stated.

# **Supplemental information and discussion**

Obesity is a major risk factor for T2D. The causative role of obesity in T2D was supported by the fact that many patients with T2D were overweight/obese [30] and that the risk of T2D could be substantially reduced by weight reduction. That said, not all obese individuals developed T2D [31], suggesting additional factors were needed for T2D to manifest. Abdominal fat comprises of two types of adipose tissue, SAT and VAT which differs from each other in molecular, cellular, anatomical, physiological, clinical, and prognostic features [32]. In a study of 726 adipose tissue samples, SAT and VAT showed differential tissue-specific gene expression, with upregulation of *HOXC* cluster and downregulation of *HOXB* cluster in SAT compared with that expressed in VAT [33]. While SAT consists predominantly of mature adipocytes for energy storage, the colocalization of premature fat cells and macrophages in VAT is associated with release of inflammatory cytokines, glucose tolerance, and lipolysis mediated insulin resistance, the main clinical and diagnostic features of T2D [34]. Given these different characteristics between SAT and VAT, we hypothesize that analysis of gene expression and DNA methylation in SAT and VAT isolated from obese individuals with or without T2D may reveal underlying mechanisms of T2D associated with obesity.

We collected SAT and VAT samples from a cohort of obese individuals, with or without T2D, who underwent metabolic surgery. As global changes in gene expression of T2D were modest [23], to address this limitation, we designed an integratome analysis that combined tissue transcriptomes with DNA methylomes and incorporated tissue-specific regulatory networks from public databases to produce an interactome for identifying molecular biomarkers linking T2D with obesity with cross-validation using public databases.

The 5’-3’ bias of all RNA samples showed no bias in T2D and control as well as SAT and VAT samples separately (Supplementary Figure S1). Transcriptome data from different tissues were analyzed separately. Age was the only significant confounder (*p<*0.01) which was adjusted in the analyses. Fewer T2D-associated DEGs were detected in adipose tissue compared with other diseases [23], particularly in SAT. Although no significantly enriched pathways were identified for the common upregulated genes, KEGG pathway analysis of the common downregulated genes revealed associations with impaired cell metabolism, decreased biosynthesis of antibiotics as well as inhibited fatty acid and amino acid degradation (Figure 1D) in obese individuals with T2D. Annotation enrichment analysis of the tissue-specific DEGs revealed their roles in different functional pathways (Figure 1C–D). Several pathways related to RNA splicing and transport, protein synthesis and inflammation were upregulated only in T2D-VAT. Some examples included the TNF signaling pathway and cytokine-cytokine receptor interactions. In addition to impaired cell metabolism, pathways related to cell growth including mTOR, ErbB, VEGF signaling, and adipocyte lipolysis and insulin signaling were inhibited in T2D-VAT.

No bias was detected in the efficiency of staining, hybridization, extension, and bisulfite conversion steps, as well as monitoring allele-specific extension (Supplementary Figure S1). As described in Material and Methods, during pre-processing of methylome data, 17,371 probes were removed in Step 1, 2,069 probes were removed in Step 2 and 2,970 and 18,980 probes were sequentially removed in Step 3. A total of 825,505 normalized probes against the entire adipose tissue methylome profile were used for analysis. We analyzed the average methylation levels per probe in T2D and control as well as SAT and VAT samples separately.

Among the 19 and 31 T2D-DMRs (p<0.05, FDR<0.2, Supplementary Table S5) in SAT and VAT, we discovered a novel hypomethylated region in the promoter of *LCLAT1* (lysocardiolipin acyltransferase) (Figure 3B) implicated in cardiolipin remodeling, a key component of the inner member of mitochondria. The promoter (TSS1500) region of *LCLAT1* showed a consistent pattern of hypomethylation in both T2D-VAT and T2D-SAT samples. The average methylation levels decreased from 0.6 in the control samples to 0.3 in the T2D samples. According to ENCODE data, when occupied by a range of TFs, this hypomethylated region could lead to H3K27ac chromatin modification (Figure 3B). ChromHMM analysis revealed that this region is an active promoter chromatin state in blood, breast, embryonic stem cell, liver, lung, muscle, and skin tissue (Figure 3B), representing facilitating TF binding and activating gene expression. We also found a novel tissue-specific hypermethylated region associated with *HOXA3* (Homeobox-leucine zipper protein), an anti-inflammatory TF. Hypermethylation was seen across the entire 5’UTR in T2D-SAT (Figure 3C). RNA-Seq analysis showed consistently lower levels of *HOXA3* mRNA in T2D-SAT (Figure 3C), supporting the hypothesis that *HOXA3* hypermethylation might lead to repression of *HOXA3* transcription.

Then, we discovered epigenetically dysregulated gene modules in adipose tissues and their associations with T2D. Three and five modules were detected for SAT and VAT, respectively (Figure 4A). We found increased expression of *ATF4* in both T2D-SAT and T2D-VAT (*p=*3.58e-02 in T2D-SAT; *p*>0.1 in T2D-VAT), consistent with the role of the protein *ATF4* encoded in glucose metabolism through inhibition of insulin secretion [35] (Supplementary Figure S3A, S3C). Other well-known T2D-related genes were also identified in both T2D-SAT and T2D-VAT FEMs, including *CNR1*, *HSD11B1*, and *IL1RL1* (Figure 3C, 3E, S3A, S3E).

Although the aforementioned genes were similarly regulated in T2D-SAT and T2D-VAT, some genes were divergently expressed/methylated in the two adipose tissues. Such genes included *ELF1* (Figure 3E, S3C), *NR3C1* (Figure 3E, S3E), *SPI1* (Supplementary Figure S3A, S3C)*, SREBF1* (Supplementary Figure S3A, S3E), and *USF1* (Supplementary Figure S3A, S3E). The TF USF1 regulates the expression of genes involved in glucose and lipid metabolism and genetic variations of USF1 had been associated with risk of T2D in the Chinese population [36]. In our analysis, we observed upregulated *USF1* expression (*p=*0.09) with decreased DNA methylation levels in T2D-SAT (Supplementary Figure S3A), which might lead to dysfunction of glucose and lipid metabolism. In the T2D-VAT, there was downregulation *USF1* expression (*p=*0.12) without any accompanying changes in methylation (Supplementary Figure S3E), suggesting that differential tissue-specific expression, rather than methylation, might contribute to development of T2D.

In T2D-VAT, we identified 5 modules (Figure 4A, S3B–F) involving 82 genes, 22 of which (26.8%) showed moderate differential expression (*p<*0.05). Circadian rhythm-associated genes were enriched among the 82 genes (FDR*=*8.54e-02), including *ARNTL*, *BHLHE40*, and *BHLHE41*. Disruption of circadian rhythm had been implicated in T2D, in part through loss and dysfunction of β-cells [37]. Among the 18 genes shared by the T2D-VAT modules, *TFEB* (Figure 4A) was numerically downregulated in 4 T2D-VAT modules (Supplementary Figure S3C-F) (*p=*0.11), which could lead to impaired adipogenesis in white adipose tissue [38]. Other genes were associated with T2D, such as *DDR2* (Supplementary Figure S3B) and *EGR3* (Supplementary Figure S3C).

Three modules were detected in T2D-SAT using the SAT-specific regulatory network, encompassing 55 genes (Figure 4A). Human T-cell leukemia virus type 1 (HTLV-I) infection-associated genes were enriched (FDR*=*5.75e-02), including *ATF4*, *EGR1*, *NFKB2*, *SPI1*, and *WNT5A*, suggesting dysregulated inflammatory responses in obese T2D individuals. Overall, 16 of the 55 genes (29.1%) showed moderately differential expression (*p<*0.05), some of which were related to T2D. Other differentially expressed T2D-related genes albeit not significant were also detected in these modules. One such gene was *PYGL,* glycogen phosphorylase, for which a small-molecule was developed for treatment of T2D [39].

The *HOX* genes was enriched in one of the T2D-SAT modules (*p=* 6.85e-08; hypergeometric test) (Figure 4C–D) which included 21 genes, 11 of which were TFs and five of which were *HOX* family genes (*HOXA10*, *HOXC10*, *HOXD9*, *MEOX2*, and *PRRX1*). The upregulation of *PRRX1* (*p=*4.26e-02) in T2D-SAT might promote TGFβ signaling and suppress *PPARγ* (*p=*1.06e-02) resulting in inhibition of adipogenesis, adipocyte hypertrophy, ectopic fat accumulation, inflammation, insulin resistance and T2D [40]. Due to the mediating roles of PPARs in adipocyte biology and inflammation, they were considered as potential therapeutic targets [41]. The downregulation of *HOXD9* (*p=*1.89-e02) could also contribute towards ectopic lipid accumulation [42] with impaired insulin signaling. The only common target of the 5 *HOX* family genes (including *PRRX1* and *HOXD9*) in this *HOX* gene family-enriched module was the TF *TLR4* (toll-like receptor 4), which was both hypomethylated and upregulated (*p=*7.35e-02). The toll-like receptor 4 (TLR4) signaling pathway was strongly implicated in obesity-induced inflammation [43]. Reduced methylation of *JNK1* had been reported in obese individuals [44] with increased JNK activity repressing *Meox2* during epithelial-to-mesenchymal transition [45]. While the regulatory relationship between *JNK* and *MEOX2* in T2D required further elucidation, increased expression of *JNK1* could increase the expression of pro-inflammatory cytokines associated with obesity [46]. *HSD11B1* (*p=*0.12) and *MME* (*p=*0.12), the common targets of *HOXA10*, *HOXC10*, and *HOXD9*, were hypomethylated and upregulated in the T2D-SAT. Genetic polymorphisms of *HSD11B1* had been associated with T2D [47], while downregulation of MME in subcutaneous preadipocytes increased inflammatory responses with increased basal insulin signalling [48]. *APCDD1*, another common target of the *HOX* genes *HOXC10*, *HOXD9*, and *MEOX2*, was hypermethylated and downregulated (*p=*2.07e-02) in T2D-SAT. Experimentally, silencing of APCDD1 inhibited expression of adipocyte differentiation markers, including *C/EBPα* and *PPARγ*, in 3T3-L1 preadipocytes [49]. Reduced expression of *APCDD1*, *C/EBPα*, and *PPARγ* had been reported in SAT from obese individuals compared to healthy controls [49]. The TF *SPI1* was hypomethylated and upregulated in T2D-SAT (*p=*7.88e-02) and might account for the upregulation of *JNK1* (*p=*6.01e-02). These could lead to obesity-induced inflammation and reduced genomic binding of non-coding RNAs to PPARγ with reduced adipogenesis [50]. In human peripheral blood mononuclear cells, human intestinal epithelial cells, and human monocytic cell lines, *SPI1* had been shown to regulate *TLR4* expression [51], while in follicular dendritic cells, *SPI1* was co-expressed with *PRRX1* [52]. The cooperative expression of *APCDD1*, *PRRX1* and *SPI1* might inhibit PPARγ-mediated adipocyte differentiation and adipogenesis, while cooperative expression of *HOXD9*, *MME*, *SPI,* and *TLR4* might impair insulin signaling and secretion accompanied by obesity-induced inflammatory responses. We identified a 5’UTR of HOXA3 as a novel T2D-SAT-specific hypermethylated region (Figure 3C) which was related to all HOXA3-regulated genes (Figure 4A), supporting their roles in the epigenetic regulation in T2D (Figure 4D).

Among the three modules identified in T2D-SAT, we detected a novel SAT-specific module associated with T2D (Figure 4E–F). Within this module, we identified a potential regulatory role of *NR3C1*. Although we only detected slight downregulation of *NR3C1*, which encoded adipocyte glucocorticoid receptor (GR)*,* the reduced GR in adipocytes could promote diet-induced inflammation [53]. Our analysis also suggested that *NR3C1* regulated *ACACB*, *DEFB132*, *FBR1* and *IL1R1.* Upregulation (*p=*2.30e-02) and hypomethylation (*p=*0.12) of *IL1RL1* could reduce adipocyte differentiation [54]. Although no significant differential expression was detected for *FBN1*, the methylation levels of this gene were increased (*p=*0.11) in T2D-SAT. This gene was a regulator of immune and inflammatory pathways and genetic mutations of this gene had been associated with failure of adipocyte differentiation [55]. We also identified reduced expression of *DEFB132* (*p=*9.10e-02) in T2D-SAT, while data from GTEx indicated overexpression of this gene in SAT of healthy subjects. Another target of *NR3C1*, *ACACB,* a key regulator in the fatty acid oxidation pathway, was downregulated (*p=*3.23e-06) with a trend towards hypermethylation (*p=*0.63) in T2D-SAT. Genetic polymorphisms of *ACACB* were associated with obesity and T2D [56]. Here, *ACACB* was regulated by *ELF1* and *TFCP2*. In T2D-SAT, *TFCP2* was downregulated (*p=*3.09e-02), which had been associated with impaired glucose metabolism, T2D, and elevated HbA1c levels [57]. *ELF1* was hypomethylated and upregulated (*p=*6.71e-02) in T2D-VAT. In myeloid cell lines, *ELF1* was reciprocally activated by *SPI1* [58]. Another target of *ELF1*, the Rap1-GTP-Interacting Adaptor Molecule (*APBB1IP*), regulated by active Rap1 in the RAP1 signaling pathway, was a modulator of T cell responses. Gene co-expression and enrichment analyses also reported a relationship between *APBB1IP* and immunological responses [59]. These novel findings were confirmed by qPCR validation where the expression levels of *ACACB*, *ELF1*, *IL1RL1* and *SPI1* were replicated in additional T2D-SAT samples (Supplementary Figure S4). Taken together, the cooperative expression of these modular genes, especially those coding for *APBB1IP*, *ACACB*, *DEFB132* and *FBN1*, might provide a novel epigenetic pathway regulating insulin signaling through adipocyte differentiation and inflammatory responses in obese patients with T2D (Figure 4F).

To confirm the reproducibility of the 161 genes identified across the different modules and DMRs (Figure 5A), we used external databases including comorbidity, druggability, eQTL, trans-ethnic GWAS, TFBSs and TFs. We also used the T2Di which was constructed by our group using transcriptomic data derived from peripheral blood cells in patients with young-onset T2D [23] (Figure 5B, Supplementary Table S6). The 161 biomarkers (74 from T2D-SAT and 111 from T2D-VAT) were identified from 118 module-associated genes and 43 DMRs. Of these, 24 biomarkers were identified in both tissues. Overall, 73.9% of biomarkers (119/161) were supported by at least one external dataset and 48.4% (78/161) were identified as TFs. Among these TFs, 51.4% were T2D-SAT biomarkers (38/74) and 50.5% were T2D-VAT biomarkers (56/111), with 16 of them shared by both tissues. Genes involved in the pathogenesis of multiple diseases tend to appear in different databases of symptoms and comorbidities [60]. Our 161 novel biomarkers were enriched in 45 diseases (*p<*0.01, hypergeometric test) (Supplementary Table S7), including cardiovascular (*p=*2.98e-08), hemic/lymphatic (*p=*1.93e-06), and immune (*p=*1.24e-05) systems. Among these biomarkers, 19 disease-related genes were represented by T2D-SAT biomarkers (19/74, 25.7%) and 17 disease-related genes by T2D-VAT biomarkers (17/111, 15.3%). Five genes were shared by the T2D-SAT and T2D-VAT biomarkers (*ELF1, IL1RL1, NFKB2, USF1,* and *ZFP57*). Furthermore, 25.7% of the biomarkers identified in T2D-SAT (19/74), and 26.1% of those identified in T2D-VAT (29/111) were identified as potential drug targets, with seven of these shared by both tissues (*ALDH1L2, CNR1, HSD11B1, IL1RL1, NFKB2, NR3C1* and *SREBF2*). We matched eQTL signals with trans-ethnic GWAS associations using a Sherlock statistical framework to validate our 161 biomarkers. Of these, 14.9% were identified in T2D-SAT (11/74) and 17.1%, in T2D-VAT (19/111), with five overlapping. Finally, four T2D-SAT biomarkers and three T2D-VAT biomarkers also appeared in the T2Di. Notably, the genes shared by T2D-SAT, T2D-VAT and the T2Di were *SPI1* and *SREBF1*. *SPI1* is a well-known TF implicated in adipogenesis and insulin signaling. Genetic polymorphisms of *SREBF1* were associated with obesity and T2D [61]. Our integrated analysis identified *NRCAM, ZBTB7B* and *TFEB* in our T2D-SAT and T2D-VAT modules which were also present in the T2Di [23] based on transcriptome analysis of PBMC in patients with young-onset T2D [23].

The study revealed perturbations in adipogenesis, inflammatory and insulin signaling pathways in obese individuals with T2D, however, it is important to recognize some limitations in the study. First, the small sample size in each condition may lead to a higher variability in the results. To improve the reproducibility, we used 47 additional samples to validate the expression of various modular genes, while further single cell analysis and validations of RNA expression may bring other valuable insights. Second, medications have been prescribed for most of the T2D patients, which cannot be well adjusted in this study. However, due to the ethical principles involving human subjects, it is not possible to take off medical treatment from the T2D patients. Therefore, the correlation between the T2D status and medication intake could not be avoided in this study. Third, coverage difference between the whole transcriptome sequencing and DNA methylation array might bring bias in the integratome analysis. Although the FEM method was developed and validated on the RNA-Seq and methylation array data, sequencing-based methylation analysis, instead of array-based methylation analysis, should be used to ensure a better genomic coverage. In addition to transcriptome, methylome and interactome, other omics datasets such as genome, proteome and metabolome could be integrated in the future study. Nevertheless, compared to individual gene or regulatory relationships, integratome analysis can reveal new insights in complex diseases such as diabetes and obesity in discovering and confirming causal biological pathways, associated biomarkers, or potential drug targets, especially those features that are not detectable by single omics approach.

# **Abbreviations**

DEG, differentially expressed gene; DMR, differentially methylated region; eQTL, expression quantitative trait loci; FDR, false discovery rate; FPKM, Fragments Per Kilobase of transcript per Million mapped reads; GO, Gene Ontology; GTEx, Genotype-Tissue Expression project; GWAS, genome-wide association studies; HOX, homeobox; KEGG, Kyoto Encyclopedia of Genes and Genomes; PC, principal component; PCA, principal component analysis; RIN, RNA integrity number; SAT, subcutaneous adipose tissue; T2D, type 2 diabetes; T2Di, T2D interactome; TF, transcription factor; TFBS, transcription factor binding site; TSS, transcription start site; VAT, visceral adipose tissue.

# **References**

1. Bolger, A.M., M. Lohse, and B. Usadel, *Trimmomatic: a flexible trimmer for Illumina sequence data.* Bioinformatics, 2014. **30**(15): p. 2114-20.

2. Dobin, A., et al., *STAR: ultrafast universal RNA-seq aligner.* Bioinformatics, 2013. **29**(1): p. 15-21.

3. Konstantin, O., et al., *Qualimap 2: advanced multi-sample quality control for high-throughput sequencing data*. Bioinformatics, 2016. **32**(2):292-4.

4. Trapnell, C., et al., *Differential gene and transcript expression analysis of RNA-seq experiments with TopHat and Cufflinks.* Nat Protoc, 2012. **7**(3): p. 562-78.

5. Ritchie, M.E., et al., *limma powers differential expression analyses for RNA-sequencing and microarray studies.* Nucleic Acids Res, 2015. **43**(7): p. e47.

6. Kanehisa, M., et al., *KEGG: new perspectives on genomes, pathways, diseases and drugs.* Nucleic Acids Res, 2017. **45**(D1): p. D353-D361.

7. *Expansion of the Gene Ontology knowledgebase and resources.* Nucleic Acids Res, 2017. **45**(D1): p. D331-D338.

8. Dennis, G., Jr., et al., *DAVID: Database for Annotation, Visualization, and Integrated Discovery.* Genome Biol, 2003. **4**(5): p. P3.

9. Jakubek, Y.A. and D.J. Cutler, *A model of binding on DNA microarrays: understanding the combined effect of probe synthesis failure, cross-hybridization, DNA fragmentation and other experimental details of affymetrix arrays.* BMC Genomics, 2012. **13**: p. 737.

10. Pidsley, R., et al., *A data-driven approach to preprocessing Illumina 450K methylation array data.* BMC Genomics, 2013. **14**: p. 293.

11. Xu, Z., et al., *ENmix: a novel background correction method for Illumina HumanMethylation450 BeadChip.* Nucleic Acids Res, 2016. **44**(3): p. e20.

12. Teschendorff, A.E., et al., *A beta-mixture quantile normalization method for correcting probe design bias in Illumina Infinium 450 k DNA methylation data.* Bioinformatics, 2013. **29**(2): p. 189-96.

13. Tian, Y., et al., *ChAMP: updated methylation analysis pipeline for Illumina BeadChips.* Bioinformatics, 2017. **33**(24): p. 3982-3984.

14. Jaffe, A.E., et al., *Bump hunting to identify differentially methylated regions in epigenetic epidemiology studies.* Int J Epidemiol, 2012. **41**(1): p. 200-9.

15. Ernst, J. and M. Kellis, *ChromHMM: automating chromatin-state discovery and characterization.* Nat Methods, 2012. **9**(3): p. 215-6.

16. Marbach, D., et al., *Tissue-specific regulatory circuits reveal variable modular perturbations across complex diseases.* Nat Methods, 2016. **13**(4): p. 366-70.

17. Kheradpour, P., et al., *Systematic dissection of regulatory motifs in 2000 predicted human enhancers using a massively parallel reporter assay.* Genome Res, 2013. **23**(5): p. 800-11.

18. Lizio, M., et al., *Gateways to the FANTOM5 promoter level mammalian expression atlas.* Genome Biol, 2015. **16**: p. 22.

19. ENCODE Project Consortium. *An integrated encyclopedia of DNA elements in the human genome.* Nature, 2012. **489**(7414): p. 57-74.

20. GTEx Consortium. *Human genomics. The Genotype-Tissue Expression (GTEx) pilot analysis: multitissue gene regulation in humans.* Science, 2015. **348**(6235): p. 648-60.

21. Roadmap Epigenomics Consortium. *Integrative analysis of 111 reference human epigenomes.* Nature, 2015. **518**(7539): p. 317-330.

22. Jiao, Y., M. Widschwendter, and A.E. Teschendorff, *A systems-level integrative framework for genome-wide DNA methylation and gene expression data identifies differential gene expression modules under epigenetic control.* Bioinformatics, 2014. **30**(16): p. 2360-6.

23. Li, J.W., et al., *Interactome-transcriptome analysis discovers signatures complementary to GWAS Loci of Type 2 Diabetes.* Sci Rep, 2016. **6**: p. 35228.

24. Menche, J., et al., *Disease networks. Uncovering disease-disease relationships through the incomplete interactome.* Science, 2015. **347**(6224): p. 1257601.

25. Griffith, M., et al., *DGIdb: mining the druggable genome.* Nat Methods, 2013. **10**(12): p. 1209-10.

26. Nica, A.C., et al., *The architecture of gene regulatory variation across multiple human tissues: the MuTHER study.* PLoS Genet, 2011. **7**(2): p. e1002003.

27. Mahajan, A., et al., *Genome-wide trans-ancestry meta-analysis provides insight into the genetic architecture of type 2 diabetes susceptibility.* Nat Genet, 2014. **46**(3): p. 234-44.

28. He, X., et al., *Sherlock: detecting gene-disease associations by matching patterns of expression QTL and GWAS.* Am J Hum Genet, 2013. **92**(5): p. 667-80.

29. Ashburner, M., et al., *Gene ontology: tool for the unification of biology. The Gene Ontology Consortium.* Nat Genet, 2000. **25**(1): p. 25-9.

30. Eckel, R.H., et al., *Obesity and type 2 diabetes: what can be unified and what needs to be individualized?* J Clin Endocrinol Metab, 2011. **96**(6): p. 1654-63.

31. Wilding, J.P., *The importance of weight management in type 2 diabetes mellitus.* Int J Clin Pract, 2014. **68**(6): p. 682-91.

32. Ibrahim, M.M., *Subcutaneous and visceral adipose tissue: structural and functional differences.* Obes Rev, 2010. **11**(1): p. 11-8.

33. Jinsoo A., et al., *Integrative Analysis Revealing Human Adipose-Specific Genes and Consolidating Obesity Loci.* Sci Rep, 2019. **9**(1):3087.

34. Maggie S. B., et al., *Contribution of adipose tissue inflammation to the development of type 2 diabetes mellitus.* Compr Physiol, 2018. **9**(1): 1–58.

35. Yoshizawa, T., et al., *The transcription factor ATF4 regulates glucose metabolism in mice through its expression in osteoblasts.* J Clin Invest, 2009. **119**(9): p. 2807-17.

36. M C Y Ng, et al., *The linkage and association of the gene encoding upstream stimulatory factor 1 with type 2 diabetes and metabolic syndrome in the Chinese population.* Diabetologia, 2005. **48**(10): p. 2018-24.

37. Kurose, T., D. Yabe, and N. Inagaki, *Circadian rhythms and diabetes.* J Diabetes Investig, 2011. **2**(3): p. 176-7.

38. Salma, N., et al., *Tfe3 and Tfeb Transcriptionally Regulate Peroxisome Proliferator-3 Receptor gamma2 Expression in Adipocytes and Mediate Adiponectin and Glucose Levels in Mice.* Mol Cell Biol, 2017. **37**(15).

39. Favaro, E., et al., *Glucose utilization via glycogen phosphorylase sustains proliferation and prevents premature senescence in cancer cells.* Cell Metab, 2012. **16**(6): p. 751-64.

40. Ehrlund, A., et al., *Transcriptional Dynamics During Human Adipogenesis and Its Link to Adipose Morphology and Distribution.* Diabetes, 2017. **66**(1): p. 218-230.

41. Stienstra, R., et al., *PPARs, Obesity, and Inflammation.* PPAR Res, 2007. **2007**: p. 95974.

42. Wan, Y., et al., *Maternal PPAR gamma protects nursing neonates by suppressing the production of inflammatory milk.* Genes Dev, 2007. **21**(15): p. 1895-908.

43. Rogero, M.M. and P.C. Calder, *Obesity, Inflammation, Toll-Like Receptor 4 and Fatty Acids.* Nutrients, 2018. **10**(4).

44. Remely, M., et al., *Microbiota and epigenetic regulation of inflammatory mediators in type 2 diabetes and obesity.* Benef Microbes, 2014. **5**(1): p. 33-43.

45. Sahu, S.K., et al., *JNK-dependent gene regulatory circuitry governs mesenchymal fate.* EMBO J, 2015. **34**(16): p. 2162-81.

46. Leavy, O., *The JNK diet.* Nat Rev Immunol, 2007. **7**: p. 918-919.

47. Devang, N., et al., *Association of HSD11B1 gene polymorphisms with type 2 diabetes and metabolic syndrome in South Indian population.* Diabetes Res Clin Pract, 2017. **131**: p. 142-148.

48. Ramirez, A.K., et al., *Membrane metallo-endopeptidase (Neprilysin) regulates inflammatory response and insulin signaling in white preadipocytes.* Mol Metab, 2019. **22**: p. 21-36.

49. Yiew, N.K.H., et al., *A novel role for the Wnt inhibitor APCDD1 in adipocyte differentiation: Implications for diet-induced obesity.* J Biol Chem, 2017. **292**(15): p. 6312-6324.

50. Dispirito, J.R., et al., *Pruning of the adipocyte peroxisome proliferator-activated receptor gamma cistrome by hematopoietic master regulator PU.1.* Mol Cell Biol, 2013. **33**(16): p. 3354-64.

51. Lichtinger, M., et al., *Transcription factor PU.1 controls transcription start site positioning and alternative TLR4 promoter usage.* J Biol Chem, 2007. **282**(37): p. 26874-83.

52. Mabbott, N.A., et al., *Expression of mesenchyme-specific gene signatures by follicular dendritic cells: insights from the meta-analysis of microarray data from multiple mouse cell populations.* Immunology, 2011. **133**(4): p. 482-98.

53. Desarzens, S. and N. Faresse, *Adipocyte glucocorticoid receptor has a minor contribution in adipose tissue growth.* J Endocrinol, 2016. **230**(1): p. 1-11.

54. Challa, T.D., et al., *Regulation of De Novo Adipocyte Differentiation Through Cross Talk Between Adipocytes and Preadipocytes.* Diabetes, 2015. **64**(12): p. 4075-87.

55. Davis, M.R., et al., *Expression of FBN1 during adipogenesis: Relevance to the lipodystrophy phenotype in Marfan syndrome and related conditions.* Mol Genet Metab, 2016. **119**(1-2): p. 174-85.

56. Riancho, J.A., et al., *Association of ACACB polymorphisms with obesity and diabetes.* Mol Genet Metab, 2011. **104**(4): p. 670-6.

57. Roberts, R.O., et al., *Diabetes and elevated hemoglobin A1c levels are associated with brain hypometabolism but not amyloid accumulation.* J Nucl Med, 2014. **55**(5): p. 759-64.

58. Calero-Nieto, F.J., et al., *Transcriptional regulation of Elf-1: locus-wide analysis reveals four distinct promoters, a tissue-specific enhancer, control by PU.1 and the importance of Elf-1 downregulation for erythroid maturation.* Nucleic Acids Res, 2010. **38**(19): p. 6363-74.

59. Ashbrook, D.G., S. Cahill, and R. Hager, *A Cross-Species Systems Genetics Analysis Links APBB1IP as a Candidate for Schizophrenia and Prepulse Inhibition.* Front Behav Neurosci, 2019. **13**: p. 266.

60. Lee, D.S., et al., *The implications of human metabolic network topology for disease comorbidity.* Proc Natl Acad Sci U S A, 2008. **105**(29): p. 9880-5.

61. Eberle, D., et al., *SREBF-1 gene polymorphisms are associated with obesity and type 2 diabetes in French obese and diabetic cohorts.* Diabetes, 2004. **53**(8): p. 2153-7.

# **Attachment**

library(FEM)

FemModShow1 <-function (mod, name, fem.o, mode = "Integration")

{

edgeweight = fem.o$ew

adjacency = fem.o$adj

mycircle <- function(coords, v = NULL, params) {

vertex.color <- params("vertex", "color")

if (length(vertex.color) != 1 && !is.null(v)) {

vertex.color <- vertex.color[v]

}

vertex.size <- 1/200 * params("vertex", "size")

if (length(vertex.size) != 1 && !is.null(v)) {

vertex.size <- vertex.size[v]

}

vertex.frame.color <- params("vertex", "frame.color")

if (length(vertex.frame.color) != 1 && !is.null(v)) {

vertex.frame.color <- vertex.frame.color[v]

}

vertex.frame.width <- params("vertex", "frame.width")

if (length(vertex.frame.width) != 1 && !is.null(v)) {

vertex.frame.width <- vertex.frame.width[v]

}

mapply(coords[, 1], coords[, 2], vertex.color, vertex.frame.color,

vertex.size, vertex.frame.width, FUN = function(x,

y, bg, fg, size, lwd) {

symbols(x = x, y = y, bg = bg, fg = fg, lwd = lwd,

circles = size, add = TRUE, inches = FALSE)

})

}

realgraph = graph.adjacency(adjacency, mode = "undirected")

E(realgraph)$weight = edgeweight

edge.width.v = E(realgraph)$weight

idxbw02 = which(edge.width.v <= 2 && edge.width.v > 0)

idxbw25 = which(edge.width.v > 2 && edge.width.v < 5)

idxbw510 = which(edge.width.v >= 5 && edge.width.v < 10)

idxgt10 = which(edge.width.v >= 10)

edge.width.v[idxbw02] = 1/4 * edge.width.v[idxbw02]

edge.width.v[idxbw25] = 1/2 * edge.width.v[idxbw25]

edge.width.v[idxbw510] = 3/4 * edge.width.v[idxbw510]

edge.width.v[idxgt10] = 10

idxlt025 = which(edge.width.v < 0.25)

edge.width.v[idxlt025] = 0.25

E(realgraph)$edgewidth = edge.width.v

mod = as.data.frame(mod)

if (mode == "Epi") {

mod[, "stat(mRNA)"] = mod[, "stat(DNAm)"]

}

else if (mode == "Exp") {

mod[, "stat(DNAm)"] = mod[, "stat(mRNA)"]

}

mod.graph = igraph::induced.subgraph(realgraph, v = as.vector(mod[,

1]))

print(mod[, 1])

mtval.v = vector()

for (i in V(mod.graph)$name) {

mtval.v = c(mtval.v, (as.vector(mod[i, "stat(DNAm)"])))

}

V(mod.graph)$mtval = mtval.v

rtval.v = vector()

for (i in V(mod.graph)$name) {

rtval.v = c(rtval.v, (as.vector(mod[i, "stat(mRNA)"])))

}

V(mod.graph)$rtval = rtval.v

print(rtval.v)

vm.color = rep(0, length(V(mod.graph)))

vr.color = rep(0, length(V(mod.graph)))

tmcolor.scheme <- maPalette(low = "yellow", high = "blue",

mid = "grey", k = 100)

trcolor.scheme <- maPalette(low = "green", high = "red",

mid = "grey", k = 100)

tmcolor.scheme[37:64] = "#BEBEBE"

tmcolor.scheme[1:36] = maPalette(low = "yellow", high = "#fcfbe3",

k = 36)

tmcolor.scheme[65:100] = maPalette(low = "#d4d4ff", high = "blue",

k = 36)

trcolor.scheme[37:64] = "#BEBEBE"

trcolor.scheme[1:36] = maPalette(low = "green", high = "#e8f9e8",

k = 36)

trcolor.scheme[65:100] = maPalette(low = "#fcdede", high = "red",

k = 36)

tmcolor.position = floor(as.numeric(V(mod.graph)$mtval)/0.04) +

51

tmcolor.position[which(tmcolor.position < 1)] <- 1

tmcolor.position[which(tmcolor.position > 100)] <- 100

vm.color = tmcolor.scheme[tmcolor.position]

V(mod.graph)$vmcolor <- vm.color

print(vm.color)

trcolor.position = floor(as.numeric(V(mod.graph)$rtval)/0.04) +

51

print(trcolor.position)

trcolor.position[which(trcolor.position < 1)] <- 1

trcolor.position[which(trcolor.position > 100)] <- 100

vr.color = trcolor.scheme[trcolor.position]

V(mod.graph)$vrcolor <- vr.color

if (mode == "Exp") {

V(mod.graph)$color <- V(mod.graph)$vrcolor

}

else {

V(mod.graph)$color <- V(mod.graph)$vmcolor

}

print(vr.color)

label.v = vector()

for (i in V(mod.graph)$name) {

label.v = c(label.v, (as.vector(mod[i, "Symbol"])))

}

V(mod.graph)$label.cex = rep(0.5, length(as.vector(V(mod.graph))))

V(mod.graph)$label.cex[which(as.vector(V(mod.graph)$name) ==

as.vector(mod[1, 1]))] = 0.8

add.vertex.shape("fcircle", clip = igraph.shape.noclip, plot = mycircle,

parameters = list(vertex.frame.color = 1, vertex.frame.width = 1))

pdf(paste(name, ".mod.pdf", sep = ""))

if (mode == "Integration") {

plot(mod.graph, layout = layout.circle,##layout.fruchterman.reingold

vertex.shape = "fcircle", vertex.frame.color = V(mod.graph)$vrcolor,

vertex.frame.width = 4, vertex.size = 10, vertex.label = label.v,

vertex.label.dist = 0.6, vertex.label.cex = V(mod.graph)$label.cex,

vertex.label.font = 3, edge.color = "grey", edge.width = E(mod.graph)$edgewidth)

colorlegend(trcolor.scheme, seq(-2, 2, 0.5), ratio.colbar = 0.3,

xlim = c(-1.55, -1.4), ylim = c(-0.5, 0), align = "r",

cex = 0.5)

colorlegend(tmcolor.scheme, seq(-2, 2, 0.5), ratio.colbar = 0.3,

xlim = c(-1.55, -1.4), ylim = c(0.5, 1), align = "r",

cex = 0.5)

text(-1.5, 0.43, c("t(DNAm)\nCore"), cex = 0.6)

text(-1.5, -0.57, c("t(mRNA)\nBorder"), cex = 0.6)

}

else if (mode == "Epi") {

plot(mod.graph, layout = layout.circle, ##layout.fruchterman.reingold

vertex.frame.color = NA, vertex.size = 10, vertex.label = label.v,

vertex.label.dist = 0.6, vertex.label.cex = V(mod.graph)$label.cex,

vertex.label.font = 3, edge.color = "grey", edge.width = E(mod.graph)$edgewidth)

colorlegend(tmcolor.scheme, seq(-2, 2, 0.5), ratio.colbar = 0.3,

xlim = c(-1.55, -1.4), ylim = c(0.5, 1), align = "r",

cex = 0.5)

text(-1.5, 0.43, c("t(DNAm)"), cex = 0.6)

}

else if (mode == "Exp") {

plot(mod.graph, layout = layout.circle, ##layout.fruchterman.reingold

vertex.frame.color = NA, vertex.size = 10, vertex.label = label.v,

vertex.label.dist = 0.6, vertex.label.cex = V(mod.graph)$label.cex,

vertex.label.font = 3, edge.color = "grey", edge.width = E(mod.graph)$edgewidth)

colorlegend(trcolor.scheme, seq(-2, 2, 0.5), ratio.colbar = 0.3,

xlim = c(-1.55, -1.4), ylim = c(-0.5, 0), align = "r",

cex = 0.5)

text(-1.5, -0.57, c("t(mRNA)"), cex = 0.6)

}

dev.off()

return(igraph.to.graphNEL(mod.graph))

}

### GenStatM1.R

GenStatM1 <- function(dnaM.m,pheno.v,sampleage,chiptype="EPIC"){

if (chiptype == "450k"){

data("probe450kfemanno")

probefemanno <- probe450kfemanno

}

else if (chiptype == "EPIC" ){

data("probeEPICfemanno")

probefemanno <- probeEPICfemanno

}

else{

print("ERROR: Please indicate correct data type!")

break

}

extractFn <- function(tmp.v, ext.idx) {

return(tmp.v[ext.idx])

}

map.idx <- match(rownames(dnaM.m), probefemanno$probeID);

probeInfo.lv <- lapply(probefemanno, extractFn, map.idx)

beta.lm <- list()

for (g in 1:6) {

group.idx <- which(probeInfo.lv[[3]] == g)

tmp.m <- dnaM.m[group.idx, ]

rownames(tmp.m) <- probeInfo.lv$eid[group.idx];

sel.idx <- which(is.na(rownames(tmp.m)) == FALSE);

tmp.m <- tmp.m[sel.idx,];

nL <- length(factor(rownames(tmp.m)));

nspg.v <- summary(factor(rownames(tmp.m)),maxsum=nL);

beta.lm[[g]] <- rowsum(tmp.m,group=rownames(tmp.m))/nspg.v;

print(paste("Done for regional gene group ", g, sep = ""))

}

unqEID.v <- unique(c(rownames(beta.lm[[2]]), rownames(beta.lm[[4]]),

rownames(beta.lm[[1]])))

avbeta.m <- matrix(nrow = length(unqEID.v), ncol = ncol(dnaM.m))

colnames(avbeta.m) <- colnames(dnaM.m)

rownames(avbeta.m) <- unqEID.v

for (gr in c(1, 4, 2)) {

avbeta.m[match(rownames(beta.lm[[gr]]), rownames(avbeta.m)),

] <- beta.lm[[gr]]

}

data.m <- avbeta.m

sampletype.f <- as.factor(pheno.v)

design.sample <- model.matrix(~0 + sampletype.f + sampleage)

colnames(design.sample) <- c(levels(sampletype.f),"age")

lmf.o <- lmFit(data.m, design.sample)

lmf.o <- eBayes(lmf.o)

cont.m <- c(-1,1,0)

lmf2.o <- contrasts.fit(lmf.o, cont.m)

bay.o <- eBayes(lmf2.o)

top.lm <- list()

c <- 1 #only one contrast

top.lm[[c]] <- topTable(bay.o, coef = c, adjust.method = "fdr", number = nrow(data.m))

return(list(top = top.lm, cont = cont.m, avbeta = avbeta.m))

}

### GenStatR1.R

GenStatR1 <- function(exp.m,pheno.v,sampleage){

if(length(grep("[a-zA-Z]",rownames(exp.m)))!=0){print("ERROR: The rownames of exp.m should be EntrezID");break}

nL <- length(factor(rownames(exp.m)));

nspg.v <- summary(factor(rownames(exp.m)),maxsum=nL);

avexp.m <- rowsum(exp.m,group=rownames(exp.m))/nspg.v;

sampletype.f <- as.factor(pheno.v)

design.sample <- model.matrix(~0 + sampletype.f + sampleage )

colnames(design.sample) <- c(levels(sampletype.f),"age")

data.m <- avexp.m

lmf.o <- lmFit(data.m, design.sample)

lmf.o <- eBayes(lmf.o)

cont.m <- c(-1,1,0)

lmf2.o <- contrasts.fit(lmf.o, cont.m)

bay.o <- eBayes(lmf2.o)

top.lm <- list()

c <- 1 #only one contrast

top.lm[[c]] <- topTable(bay.o, coef = c, adjust.method = "fdr", number = nrow(data.m))

return(list(top = top.lm, cont = cont.m, avexp = avexp.m))

}

# Take SAT as an example

# Load methylation data

dnaM.m<-read.csv("betas_sites.csv",row.names=1,header=T,sep=",")

dnaM.m<-data.matrix(dnaM.m)

dnaM.m<-dnaM.m[,-1:-4]

dnaM.m.SF<-dnaM.m[,1:19]

pheno.m.SF<-c("DM2","DM2","DM2","NoDM2","NoDM2","DM2","NoDM2","DM2","NoDM2","DM2","DM2","NoDM2","DM2","NoDM2","NoDM2","NoDM2","NoDM2","NoDM2","DM2")

conf.m.SF<-read.table("multilog_sat_meth.txt",row.names=1,header=T)

conf.m.SF<-conf.m.SF[as.character(colnames(dnaM.m.SF)),]

# Load expression data

FPKM_all<-read.table("FPKM_all_entrez_filter_0.1_SF.txt",row.names=1,header=T)

FPKM_all<-as.matrix(FPKM_all[,-1])

FPKM_all = data.matrix(FPKM_all)

FPKM_all.SF<-FPKM_all

pheno.r<-c("DM","DM","DM","DM","DM","DM","DM","DM","DM","DM","DM","NoDM","DM","NoDM","NoDM","NoDM","NoDM","NoDM","NoDM","NoDM","NoDM","NoDM","NoDM","DM","DM","DM","DM","DM","DM","DM","DM","DM","DM","DM","NoDM","NoDM","NoDM","NoDM","NoDM","NoDM","NoDM","NoDM","NoDM","NoDM") ###reversal of the orginal group

pheno.r.SF<-pheno.r[1:23]

conf.r.SF<-read.table("multilog_sat_rna.txt",row.names=1,header=T)

conf.r.SF<-conf.r.SF[as.character(colnames(FPKM_all.SF)),]

statM.o.SF <- GenStatM1(dnaM.m.SF,pheno.m.SF,conf.m.SF$Age,"EPIC")

statR.o.SF <- GenStatR1(FPKM_all.SF,pheno.r.SF,conf.r.SF$Age)

# Load tissue specific network

SF_network_raw<-read.table("SF_network_nonred.txt",header=F)

SF_network_order<-SF_network_raw[order(SF_network_raw$V3,decreasing=T),]

SF_network_top<-SF_network_order[1:10000,1:2]

G <- graph.data.frame(SF_network_top,directed=FALSE)

A <- as_adjacency_matrix(G,names=TRUE,sparse=FALSE)

intFEM.o.SF<-DoIntFEM450k(statM.o.SF,statR.o.SF,A,1,1)

intMeth.o.SF<-DoIntEpi450k(statM.o.SF,A,1)

# Generate modules

fembi.o.SF <- DoFEMbi(intFEM.o.SF, nseeds=200,gamma=0.5,nMC=5000,sizeR.v=c(1,100),minsizeOUT=10,writeOUT=TRUE,nameSTUDY="DMSF",ew.v=NULL)

library("marray")

library("corrplot")

for(m in 1:length(names(fembi.o.SF$topmod))){

FemModShow1(fembi.o.SF$topmod[[m]], name=names(fembi.o.SF$topmod)[m],fembi.o.SF)

}

write.table(statM.o.SF$top[[1]],"statM.o.SF.txt",quote=F,sep="\t")

write.table(statR.o.SF$top[[1]],"statR.o.SF.txt",quote=F,sep="\t")

write.table(fembi.o.SF$adj,"SF_adj.txt",quote=F,sep="\t")

write.table(fembi.o.SF$ew,"SF_edgeweight.txt",quote=F,sep="\t")

write.table(SF_network_top,"SF_network_top.txt",quote=F,sep="\t",rownames=F)
